# HTreeQA: Using Semi-Perfect Phylogeny Trees in Quantitative Trait Loci Study on Genotype Data

**DOI:** 10.1534/g3.111.001768

**Published:** 2012-02-01

**Authors:** Zhaojun Zhang, Xiang Zhang, Wei Wang

**Affiliations:** * Department of Computer Science, University of North Carolina at Chapel Hill, Chapel Hill, North Carolina 27599; † Department of Electrical Engineering and Computer Science, Case Western Reserve University, Cleveland, Ohio 44106

**Keywords:** phylogeny, quantitative trait loci (QTL), Mouse Collaborative Cross, Mouse Genetic Resource

## Abstract

With the advances in high-throughput genotyping technology, the study of quantitative trait loci (QTL) has emerged as a promising tool to understand the genetic basis of complex traits. Methodology development for the study of QTL recently has attracted significant research attention. Local phylogeny-based methods have been demonstrated to be powerful tools for uncovering significant associations between phenotypes and single-nucleotide polymorphism markers. However, most existing methods are designed for homozygous genotypes, and a separate haplotype reconstruction step is often needed to resolve heterozygous genotypes. This approach has limited power to detect nonadditive genetic effects and imposes an extensive computational burden. In this article, we propose a new method, HTreeQA, that uses a tristate semi-perfect phylogeny tree to approximate the perfect phylogeny used in existing methods. The semi-perfect phylogeny trees are used as high-level markers for association study. HTreeQA uses the genotype data as direct input without phasing. HTreeQA can handle complex local population structures. It is suitable for QTL mapping on any mouse populations, including the incipient Collaborative Cross lines. Applied HTreeQA, significant QTLs are found for two phenotypes of the PreCC lines, white head spot and running distance at day 5/6. These findings are consistent with known genes and QTL discovered in independent studies. Simulation studies under three different genetic models show that HTreeQA can detect a wider range of genetic effects and is more efficient than existing phylogeny-based approaches. We also provide rigorous theoretical analysis to show that HTreeQA has a lower error rate than alternative methods.

The goal of quantitative trait locus (QTL) mapping is to find strong associations representing (genomically proximal) causal genetic effects between observed quantitative traits and genetic variations. There are several mouse resources such as the Collaborative Cross (CC) (The Complex Trait Consortium 2004; Collaborative Cross Consortium 2012), Heterogeneous Stock (Valdar *et al.* 2006), and Diversity Outbred (Collaborative Cross Consortium 2012; Svenson *et al.* 2012) for large-scale association study of complex traits, among which the CC captures the most genetic and phenotypic diversity (Roberts *et al.* 2007; Aylor *et al.* 2011).

Many previous QTL mapping methods consider each genetic marker independently (Akey *et al.* 2001; Thomas 2004; Pe'er *et al.* 2006). Standard statistical tests (such as the *F*-test) are used to measure the significance of association between a phenotype and every single nucleotide polymorphism (SNP) in the genome. These single marker−based methods usually do not consider the effects of (both genotyped and ungenotyped) neighboring markers and hence may fail to discover QTL for complex traits. To address this limitation, cluster-based methods, such as HAM (Mcclurg *et al.* 2006), QHPM (Onkamo *et al.* 2002), and HapMiner (Li and Jiang 2005), have been developed. Typically the genome is partitioned into a series of intervals. For each interval, these methods first cluster samples based on the genotypes within it and then assess the statistical correlation between the clusters and the phenotype of interest. The result is sensitive to the granularity of the partition, the definition of genotype similarity, and the choice of clustering algorithms. More importantly, these methods tend to emphasize mutations as the major events that cause the differences in the DNA sequences of the samples. This may not fully represent the genetic background underlying the differences.

Phylogeny trees have been widely used to model evolutionary history among different species, subspecies, or strains (Yang *et al.* 2011). Their application in association study requires inferring an accurate global phylogeny tree from the DNA sequences (Larribe *et al.* 2002; Morris *et al.* 2002; Minichiello and Durbin 2006). This may not be feasible for the high-density markers in current QTL analysis. Some recent methods, such as Genomic Control (Devlin and Roeder 1999), EIGENSTRAT (Price *et al.* 2006), and EMMA (Kang *et al.* 2008), build global models to account for genetic effects. EMMA computes a kinship matrix to correct the effect of the population structure. Genomic Control estimates an inflation factor of the test statistics to account for the inflation problem caused by unbalanced population structure. EIGENSTRAT performs an orthogonal transformation on the genotypes using principal component analysis and then conducts the association study in this transformed space. However, the genetic background of the samples may not always be adequately captured by a global model. This is particularly true for the incipient Collaborative Cross population (PreCC). There is no significant global population stratification among the PreCC lines because each of the eight founders contributes roughly one-eighth of their entire genome (Aylor *et al.* 2011). This unique design removes the need for global population structure correction in QTL mapping.

However, *local* population structures may still exist. Because of the limited number of recombinations occurred since the founder generation, the genome of each CC line is a coarse mosaic of composed segments from the eight founders. In a genomic region, a CC line may be determined by the contribution from a single founder and none from the rest. Because the eight founders are from three subspecies, local population structure may exist in these CC lines. We have observed uneven genetic background at the chromosome level in the 184 genotyped PreCC lines, and such pattern becomes stronger when we examine at finer resolutions. (Please see *Results and Discussion* for further discussion of the local population structure in the PreCC lines.)

Local phylogeny becomes a natural choice for capturing this type of effect. Several recent methods [*e.g.,* TreeLD (Zöllner and Pritchard 2005), TreeDT (Sevon *et al.* 2006), BLOSSOC (Mailund *et al.* 2006; Besenbacher *et al.* 2009), and TreeQA (Pan *et al.* 2008, 2009)] have adopted local perfect phylogeny trees to model the genetic distance between samples. These methods examine possible groupings induced by each local phylogeny and report the ones showing strong statistical associations with the phenotype. Because these methods require a large number of statistical tests and their results are often corrected by large permutation tests, they are prone to multiple testing errors and incur significant computational burden. TreeLD and TreeDT can handle only a very small number of SNP markers and thus they are not suitable for large-scale QTL mapping. BLOSSOC is more efficient and can process the entire genome but still needs days to perform a large number of permutation tests. The recently proposed TreeQA algorithm uses several effective pruning techniques to reduce computational burden and is able to finish large permutation tests in a few hours.

A common limitation shared by all of these local phylogeny-based methods is that the perfect phylogeny trees can be only constructed from haplotypes. These methods either assume that samples are purebred (*i.e.,* no heterozygosity), which is not true for many large mammalian resources, including the PreCC lines, or that a preprocessing step *phases* each genotype into a pair of haplotypes. However, haplotype reconstruction itself is a nontrivial process that is both time-consuming (Scheet and Stephens 2006) and error-prone (Ding *et al.* 2008). Even if haplotypes are phased accurately, the two haplotypes of the same sample may be located at different branches of a phylogeny tree and will be treated as if they were independent samples in subsequent statistical tests. This may create a bias favoring additive effects and lead to spurious results. For example, consider a recessive phenotype, we use *A*/*a* to represent the majority and minority alleles at the causative locus. The local phylogeny tree built from the surrounding region has an edge corresponding to the causative SNP that separates the samples into two groups carrying *A* and *a* alleles, respectively. Each heterozygous *A*/*a* sample is phased into two haplotypes, each belonging to a different group. The group having allele *a* would have mixed phenotypes. This may weaken the power of any statistical tests and fail to detect the causative edge (Wang and Sheffield 2005, Lettre *et al.* 2007). The scenario may become even worse for phenotypes having overdominant effects on heterozygous samples.

Therefore, a natural question to ask is whether we can design a phylogeny-based QTL mapping that can be applied to unphased genotypes directly. In this article, we introduce the model of tristate semi-perfect phylogeny tree directly built from unphased genotype data and explore its utility in QTL study. Our method, HTreeQA, has the advantages of phylogeny-based methods but does not require a separate phasing step. We demonstrate via simulation studies that HTreeQA can detect a wider range of genetic effects than other alternative methods.

## Materials

### Collaborative Cross

We use the genotypes of 184 partially inbred mice from the CC lines (Aylor *et al.* 2011). On average, these mice have undergone 6.7 generations of inbreeding and have 16% heterozygosity. The genotypes at approximately 180K SNPs are collected using the mouse diversity array (Yang *et al.* 2009). The data can be accessed through the CC status website (http://csbio.unc.edu/CCstatus/index.py). We study two phenotypes. One is the white head spot, which was originally observed on one of the CC founders, WSB/EiJ. Because there are no white head-spotted mice found in F1 crosses of the CC founders, the phenotype is believed to be a recessive trait. Among the 184 mice, there are four with white head spot. Another phenotype we study is the average daily running distance for mice of 5 to 6 days old. This is a typical measurement for mouse activity. The phentotypes are supplied as supporting information, File S1.

### Synthetic data sets

The phenotype was simulated using three different models of genetic effects: additive, recessive, and overdominant (a special case of epistasis effect) models. We include the overdominant model because we observe that heterozygous individuals sometimes exhibit extreme phenotypes. This phenomenon cannot be captured by an additive or recessive model.

To simulate phenotypes, we adopt the method used in Long and Langley 1999. To simulate an additive phenotype for a given SNP, we use the following formula:

$$y_{i}=\sqrt{1-\pi }N\left(0,1\right)+Q_{i}\sqrt{\frac{\pi }{2p\left(1-p\right)}},$$

where π is the percentage of the variation attributable to the quantitative trait nucleotide, *N*(0, 1) is the standard normal distribution, and *p* is the minor allele frequency. In the additive model, *Q_i_* takes values −1, 0, and 1 for homozygous wild-type, heterozygous type, or homozygous type, respectively. For recessive and overdominant models, we use

$$y_{i}=\sqrt{1-\pi }N\left(0,1\right)+Q_{i}^{\prime }\sqrt{\frac{\pi }{2p\prime \left(1-p\prime \right)}},$$

where *p*′ is the fraction of individuals that are homozygous mutants. In a recessive model, $Q_{i}^{\prime }$ is 1 for homozygous mutant and 0 otherwise. In an overdominant model, *Q_i_* takes 1 for heterozygous mutant and 0 otherwise. All causative SNPs are removed from the genotypes before analysis. We represent results of a wide range of realistic contributions of genetic variations by testing five genetic variation settings of π: 0.05, 0.1, 0.15, 0.2, and 0.25.

We simulated genotypes of 170 independent individuals. Under each genetic effect model, we generated 100 independent test cases under each setting. In each case, there are 10,000 SNPs and one causative SNP is randomly picked among the SNPs with minor allele frequency greater than 0.15.

## Methods

### Notations

We follow the convention of using primed notation for unphased genotype data. Suppose that there are *m* individuals and *n* SNPs. We use $\left\{{S^{\prime }}_{1},{S^{\prime }}_{2},\ldots ,{S^{\prime }}_{n}\right\}$ to represent the unphased SNPs and {*S*_1_, *S*_2_, …, *S_n_*} to represent the phased SNPs. The unphased genotypes can be represented as an *m* × *n* matrix M′, where the *k*-th row corresponds to the genotype of the *k*-th individual and the *l*-th column corresponds to the *l*-th SNP marker ${S^{\prime }}_{l}$. Similarly, the 2*m* haplotypes can be represented as a 2*m* × *n* matrix M, where the 2*k*-th and (2*k* + 1)-th rows correspond to the haplotypes of the *k*-th individual. In the haplotype matrix M, we use 0 and 1 to represent the major allele and the minor allele of a SNP respectively. In the genotype matrix M′, we use 0, 1, and H to represent the homozygous major allele, the homozygous minor allele, and the heterozygous allele of a SNP, respectively. Table 1A shows an unphased genotype matrix, and Table 1B shows a phased haplotype matrix.

**Table 1:** An example of unphased data (A), its phased data (B), and its transformed result (C)

A. The unphased haplotype matrix															
Sample ID	${S^{\prime }}_{1}$	$S_{2}^{\prime }$	$S_{3}^{\prime }$	$S_{4}^{\prime }$	$S_{5}^{\prime }$	Phenotype									
A	0	0	1	1	0	10
B	0	0	1	0	1	10
C	H	1	0	0	0	2
D	H	H	0	0	0	10
E	1	1	0	0	0	2									

B. The phased haplotype matrix															
Haplotype ID	*S*_1_	*S*_2_	*S*_3_	*S*_4_	*S*_5_	Phenotype									
A1	0	0	1	1	0	10
A2	0	0	1	1	0	10
B1	0	0	1	0	1	10
B2	0	0	1	0	1	10
C1	0	1	0	0	0	2
C2	1	1	0	0	0	2
D1	0	0	0	0	0	10
D2	1	1	0	0	0	10
E1	1	1	0	0	0	2
E2	1	1	0	0	0	2									

C. The transformed genotype matrix															
ID	$S_{1}^{\prime }(0)$	$S_{1}^{\prime }(1)$	$S_{1}^{\prime }(H)$	$S_{2}^{\prime }(0)$	$S_{2}^{\prime }(1)$	$S_{2}^{\prime }(H)$	$S_{3}^{\prime }(0)$	$S_{3}^{\prime }(1)$	$S_{3}^{\prime }(H)$	$S_{4}^{\prime }(0)$	$S_{4}^{\prime }(1)$	$S_{4}^{\prime }(H)$	$S_{5}^{\prime }(0)$	$S_{5}^{\prime }(1)$	$S_{5}^{\prime }(H)$
A	**0**	**0**	0	**0**	**0**	0	**1**	1	**0**	**1**	1	**0**	**0**	0	**0**
B	**0**	**0**	0	**0**	**0**	0	**1**	1	**0**	**0**	0	**0**	**1**	1	**0**
C	**1**	**0**	1	**1**	**1**	0	**0**	0	**0**	**0**	0	**0**	**0**	0	**0**
D	**1**	**0**	1	**1**	**0**	1	**0**	0	**0**	**0**	0	**0**	**0**	0	**0**
E	**1**	**1**	0	**1**	**1**	0	**0**	0	**0**	**0**	0	**0**	**0**	0	**0**

Bold columns are selected for building the tristate semi-perfect phylogeny tree.

### Perfect phylogeny tree

An *interval* along the genome consists of a set of consecutive SNPs. It corresponds to a submatrix C_*u*,*v*_(M) of M that contains all columns between the *u*-th column and the *v*-th column. A *perfect phylogeny tree* is the tree representation of the evolution genealogy for an interval in the genome (Gusfield 1991).

#### Definition 1:

Given an interval Cu,v(M) of 2m haplotypes and n SNPs, a perfect phylogeny tree is a tree, in which the haplotype sequences are the leaves and SNPs are the edges. Given an allele of any SNP, the subgraph induced by all the nodes that carry the same allele is still a connected subtree.

**Figure 1:**
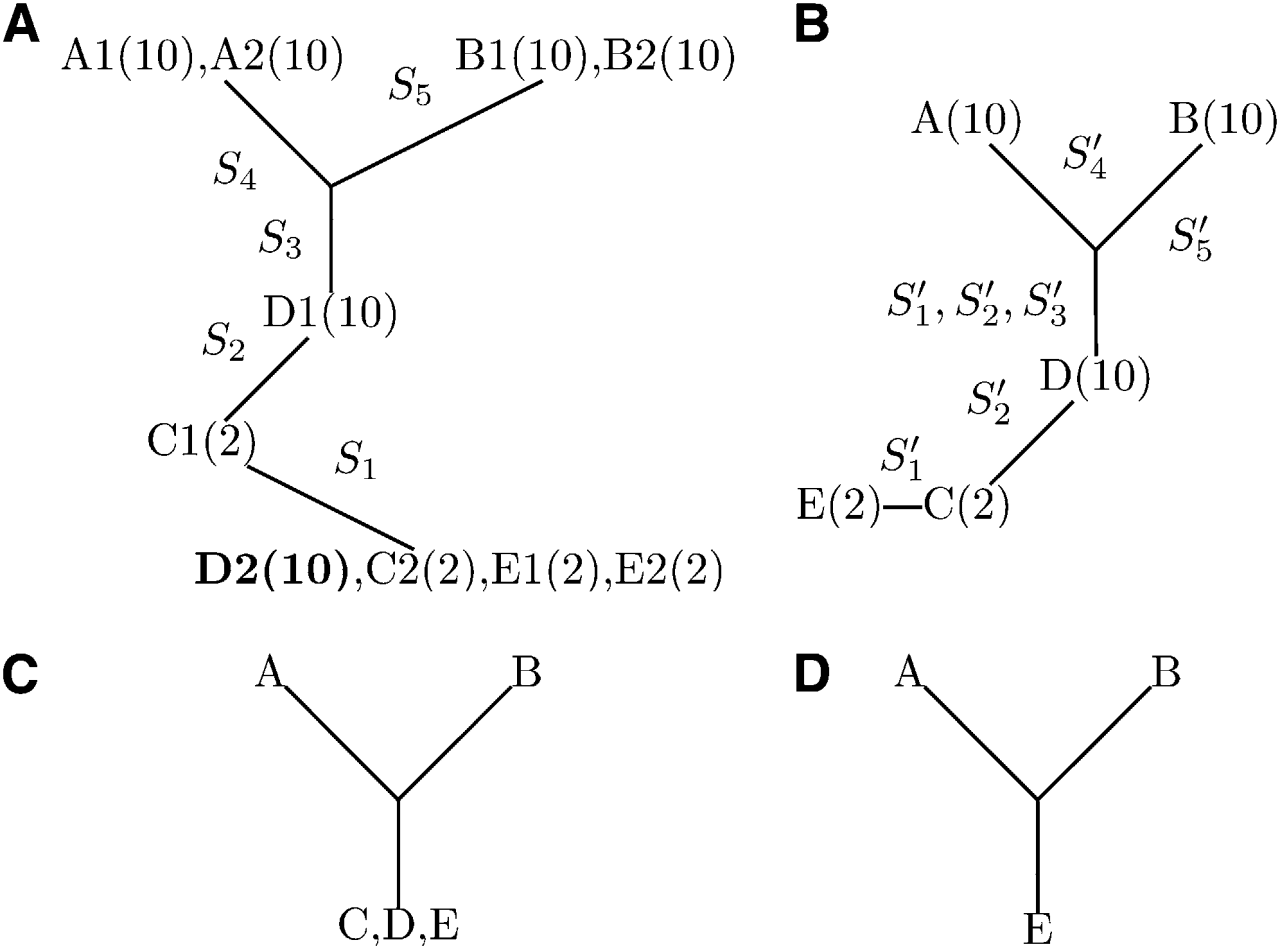
(A) is the perfect phylogeny tree generated on the phased haplotypes in Table 1B. Each node is labeled by its haplotype ID, followed by the corresponding phenotype value. (B) is a tristate semi-perfect phylogeny tree generated on the unphased genotypes in Table 1A. Each node is labeled by its sample ID followed by the corresponding phenotype value. (C) is the corresponding perfect phylogeny tree by deleting $S_{1}^{\prime }$ and $S_{2}^{\prime }$ in Table 1A, and (D) is the corresponding perfect phylogeny tree by deleting samples *C* and *D* in Table 1A.

The perfect phylogeny can be treated as an evolutionary history for the interval. Each edge represents the mutation event that derives two alleles of the corresponding SNP. All the haplotypes can be explained by the the evolutionary history without any recombination event. For example, Figure 1A shows the perfect phylogeny tree built from the haplotypes in Table 1B.

### Compatible interval

An interval C_*u*,*v*_(M) is a *compatible interval* if every pair of SNP markers in the interval pass the four-gamete test (Hudson and Kaplan 1985). That is, at most three of the four possible allele pairs {00, 01, 10, 11} appear in each pair of SNPs in the interval. This implies the existence of an evolution genealogy that can explain the evolutionary history of these two markers without recombination events, given the assumption of an infinite site model (*i.e.,* no homoplasy). For a given interval, a perfect phylogeny exists if and only if the interval is a compatible interval. If a compatible interval is not a subinterval of another compatible interval, it is called a *maximal* compatible interval.

### Tristate semi-perfect phylogeny tree

The multistate perfect phylogeny tree (Gusfield 2010) is a natural extension of the perfect phylogeny tree discussed previously. It was originally proposed to model the rare events having multiple mutations at a single locus. Because the perfect phylogeny cannot handle heterozygous site properly, we propose a novel utility of the multistate phylogeny in modeling heterozygosity in QTL mapping. By treating the heterozygous allele as the third status, a tristate phylogeny tree can be generated from a set of unphased genotypes. Because this third state is not a result of a single mutation, the tristate phylogeny tree is a relaxation of a perfect phylogeny tree.

#### Definition 2:

Given an interval $C_{u,v}\left(M\prime \right)$ of m genotypes and n SNPs, a tristate semi-perfect phylogeny tree is a tree in which the genotype sequences are the leaves and SNPs are the edges. A SNP corresponds to an edge if only two of the three possible alleles are observed and corresponds to two edges if all three alleles are observed. Given an allele of any SNP, the subgraph induced by all the nodes that carry the same allele is still a connected subtree.

### Compatibility test on genotype data

Given an interval $C_{u,v}\left(M\right)$ in the genotype matrix, we construct a binary matrix $\bar{C_{u,v}\left(M\prime \right)}$. Each column ${S^{\prime }}_{i}$ in $C_{u,v}\left(M\right)$ corresponds to three binary columns ${S^{\prime }}_{i}\left(0\right)$, ${S^{\prime }}_{i}\left(1\right)$, and ${S^{\prime }}_{i}\left(H\right)$ in $\bar{C_{u,v}\left(M\prime \right)}$. ${S^{\prime }}_{i}\left(0\right)$ is generated from ${S^{\prime }}_{i}$ by replacing every ‘H’ in ${S^{\prime }}_{i}$ by ‘1’. ${S^{\prime }}_{i}\left(1\right)$ is generated from ${S^{\prime }}_{i}$ by replacing every ‘H’ in ${S^{\prime }}_{i}$ by ‘0’. ${S^{\prime }}_{i}\left(H\right)$ is generated from ${S^{\prime }}_{i}$ by replacing every ‘H’ in ${S^{\prime }}_{i}$ by ‘1’ and ‘0’ and ‘1’ in ${S^{\prime }}_{i}$ by ‘0.’ This is equivalent to representing the ‘0,’‘1,'and ‘H’ alleles in the heterozygous ${S^{\prime }}_{i}$ by triplets (0,0,0), (1,1,0), and (1,0,1), respectively. For example, Table 1C shows the generated binary matrix $\bar{C_{u,v}\left(M\right)}$ for the genotype matrix C_*u*,*v*_(M) in Table 1A. Note that all states in $\bar{C_{u,v}\left(M\right)}$ are identical to that in C_*u*,*v*_(M′) except the ‘H’ alleles and *S*′(*H*) columns. Given an interval, the following theorem states the necessary and sufficient condition for the existence of a tri-state semi-perfect phylogeny (Dress and Steel 1992).

### Theorem 1:

Given an interval $C_{u,v}\left(M\prime \right)$ in the genotype matrix, there exists a tristate semi-perfect phylogeny, if and only if there exists a submatrix S formed by selecting two of the three columns in $\bar{C_{u,v}\left(M\prime \right)}$ for each SNP marker, and any pair of columns in S pass the four-gamete test.

An integer linear programming approach (Gusfield 2010) can be used to determine whether an interval is compatible and to compute the submatrix S. For example, in the matrix $\bar{C_{u,v}\left(M\prime \right)}$ shown in Table 1C, the columns selected for *S* are boldface. Once S is computed, a tristate semi-perfect phylogeny tree can be constructed by applying any standard perfect phylogeny tree algorithm on S. For example, Figure 1B shows the tristate semi-perfect phylogeny tree constructed from the matrix *S* in Table 1C.

If there is no heterozygous allele, each genotype will be composed of two identical haplotypes; the tristate semi-perfect phylogeny tree is identical to the perfect phylogeny tree constructed on the haplotypes. If there are some heterozygous genotypes, removing the rows or columns in the matrix containing the heterozygous alleles does not affect the remaining part of the phylogeny tree. The tree in Figure 1C shows the perfect phylogeny tree constructed on $S_{3}^{\prime },S_{4}^{\prime },S_{5}^{\prime }$ in Table 1A, which can also be derived by collapsing the three edges labeled by $S_{1}^{\prime }$ or $S_{2}^{\prime }$ in Figure 1B. If we remove nodes C and D (that have heterozygous genotypes) in Figure 1B, the resulting tree is also identical to the perfect phylogeny tree constructed on A, B, E (Figure 1D). We observe that any heterozygosity only introduces local variations in a phylogeny tree.

Another important observation can be made by comparing the perfect phylogeny tree constructed on the haplotypes to the genotype matrix. When the genotype matrix contains a small percentage of heterozygosity, the tristate semi-perfect phylogeny tree shares a substantial common structure with the perfect phylogeny tree on the haplotypes. Figure 1A shows the perfect phylogeny tree constructed on the haplotypes in Table 1B. Note that the two haplotypes (*e.g.,* D1, D2) of the same genotype (*e.g.* D) may be associated with different nodes in the tree. We will show later that this decoupling will weaken the power of detecting nonadditive genetic effects. However, this tree shares common induced subtrees with the tristate semi-perfect phylogeny tree. Removing the nodes associated with the decoupled haplotypes will result in Figure 1D, whereas collapsing edges connecting these nodes will result in Figure 1C.

### Phylogeny tree−based test

An edge in a phylogeny tree connects two disjoint subtrees. Removing *x* edges partitions the tree into *x* + 1 subtrees. For example, removing the two edges labeled with $S_{1}^{\prime }$ and $S_{2}^{\prime }$ in Figure 1B partitions genotypes into three groups {A, B, D}, {C}, and {E}.

The statistical correlation between a partition and the phenotype can be examined by the F-statistics. Assuming that for a total of *t* individuals, we have *p* groups, and the *i*th group contains *t_i_* individuals. We use *X_ij_* to represent the *i*th element in the *j*th group, ${\overset{¯}{X}}_{j}$ to represent the mean of the *j*th group, and $\overset{¯}{X}$ to represent the overall mean value. Given such a grouping of phenotype values, *G*, the F-statistics is defined as

(1) $$F\left(G\right)=\frac{\sum_{j=1}^{p}t_{j}{\left({\overset{¯}{X}}_{j}-\overset{¯}{X}\right)}^{2}}{\sum_{j=1}^{p}\sum_{i=1}^{t_{j}}{\left(X_{ij}-{\overset{¯}{X}}_{j}\right)}^{2}}.$$

The corresponding *P*-value of *F*(*G*) can be calculated in the following way. If the phenotype values from each group follow a normal distribution, an *F*-test is applied to obtain the corresponding *P*-value. Otherwise, a permutation test is needed. The *P*-value is defined as $\frac{n}{nPerm}$ where *nPerm* is the number of permutations and *n* is the number of times when the F-statistics of the permuted phenotype is larger than *F*(*G*).

We examine all possible partitions generated by removing edges in the tree. The partition that generates the most significant *P*-value is reported. The corresponding *P*-value is used as the nominal (uncorrected) *P*-value of the association between the compatible interval and the phenotype.

### Permutation test for family-wise error rate (FWER) controlling

**Figure 2:**
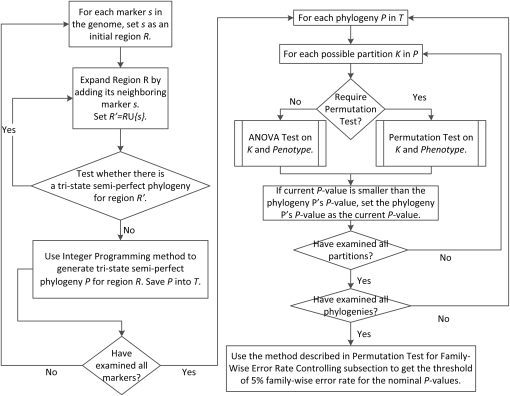
The workflow of HTreeQA. The inputs are the genotype and phenotype data. The output is a list of phylogenies and their *P*-values for measuring the association with the phenotype, and a threshold of *P*-value representing the 5% FWER.

Appropriate multiple testing correction is crucial for QTL studies. In HTreeQA, we apply the widely used permutation test to control family-wise error rate (Westfall and Young 1993; Churchill and Doerge 1994). In each permutation, the phenotype values are randomly shuffled and reassigned to individuals. For each permuted phenotype, we repeat the previously described procedure and find the smallest *P*-value. The corrected *P*-value is the proportion of the permuted data whose *P*-values are more significant than that of the original data. We refer to such a corrected *P*-value as the permutation *P*-value. The basic routine of HTreeQA is summarized in Figure 2.

### Comparison between TreeQA and HTreeQA

We outline two alternative approaches for local phylogeny-based QTL mapping methods and discuss their pros and cons.

- HTreeQA: We compute compatible intervals by using integer linear programming and construct a tristate semi-perfect phylogeny tree for each compatible interval. Then we follow the procedure described above to find significant associations.
- Running TreeQA on phased data: We first phase the genotypes using any standard phasing algorithm and then apply TreeQA on the resulting haplotypes. Each haplotype is assumed to have the same phenotype value as the original genotype.

The second approach has an inherent drawback. It decouples the two haplotypes of the same genotype. As a result, the two haplotypes may reside in remote branches of the tree, which limits the ability to test certain genetic effects in QTL mapping. For example, the phenotype in Table 1A follows a recessive model defined on $S_{2}^{\prime }$ : the phenotype is 2 for samples (C, E) having minor allele (‘1’) and is 10 for the remaining samples A, B, D (with alleles ‘0’ or ‘H’). There does not exist a set of edges in Figure 1A that can perfectly separate these two groups. (The haplotype D2 will always be in the same group as C1, E1, E2.) In contrast, the tristate semi-perfect phylogeny tree has an edge $S_{2}^{\prime }$ that perfectly separates A, B, and D from C, E. Therefore, the tristate semi-perfect phylogeny tree is more suitable for handling heterozygosity in association studies. We provide a theoretical comparison of these two approaches in Appendix 1.

## Results and Discussion

### Population structure in the PreCC lines

**Figure 3:**
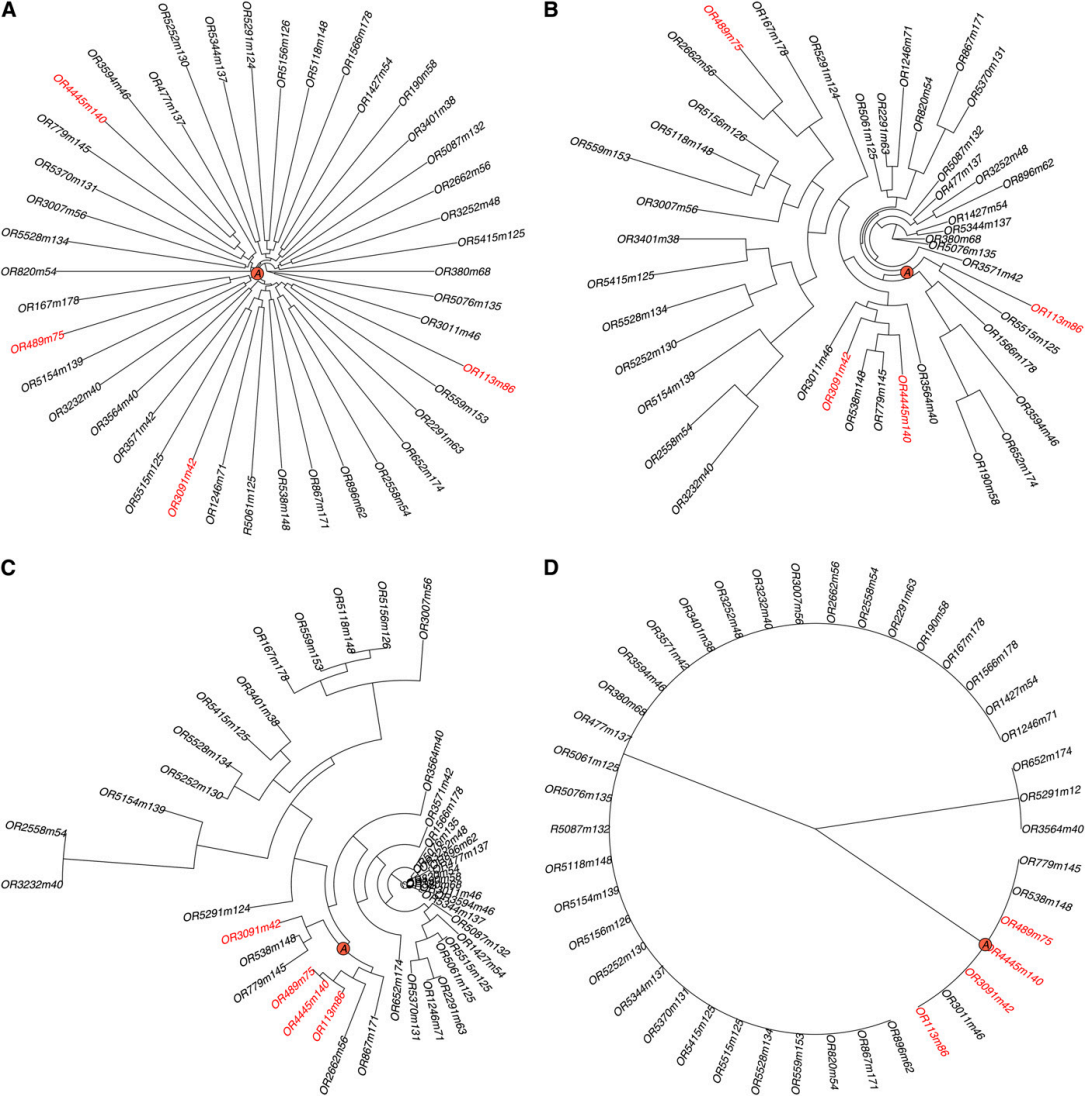
Four phylogenies of 43 randomly selected (from a total of 184) PreCC mice. The sum of the edge depth between a leaf and the origin represents the genetic distance of the corresponding mouse from the common ancestry of the 43 mice. The mice with white head spot are highlighted in red. Their nearest common ancestor is indicated by a circled “A” in each figure. In (A), the global phylogeny is balanced, and all mice are almost equally distant from each other. The phylogenies in (B) and (C) are no longer balanced, with several deep branches. The local population structure is a confounding factor that complexes the QTL analysis. The tristate semi-perfect phylogeny in (D) has the simplest structure, with an informative branch that contains all four white spot mice.

**Figure 4:**
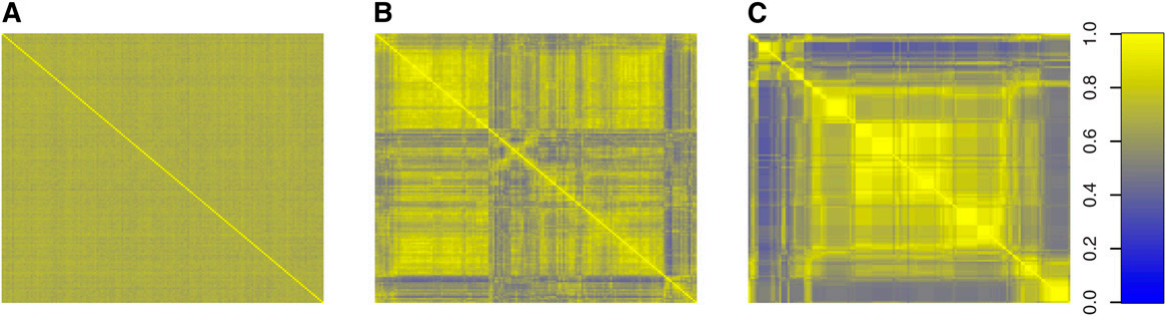
Three kinship matrices represent the genetic relatedness over the entire genome between any pair of the 184 CC mice based on the whole genome (A), the chromosome 10 (B), and the 20-Mbps interval in Chromosome 10 (C) respectively. The mice are arranged in the same order in both x and y axes. In (A), all off-diagonal entries have almost identical values, suggesting that there is no global population structure. In (B) and (C), the mice are arranged in the order of their genetic relatedness, genetically similar mice are near each other.

Population stratification is an important issue in QTL analysis. Spurious associations may be induced by the stratification if it is not addressed properly (Kang *et al.* 2008). The combinatorial breeding design of the CC yields genetically independent incipient CC lines, which ensures balanced contributions of all eight founder strains without noticeable global population stratification (Aylor *et al.* 2011). Figure 3A shows a global phylogeny tree of 43 randomly selected PreCC lines. The balanced tree structure illustrates that these mice are genetically diverse and equally distant from each other. This observation is further confirmed by the kinship matrix in Figure 4A used by EMMA for modeling genetic background (Kang *et al.* 2008). In Figure 4A, each row (column) of the kinship matrix corresponds to a CC strain. Each entry in the matrix is the kinship coefficient that represents the genetic relatedness between the two mice. We can observe that all off-diagonal entries in Figure 4A have almost identical values (around 0.8), which suggests that no significant global population stratification exists in these PreCC mice. (In Appendix 2, we provide a statistical analysis that EMMA degenerates to a standard linear model when applied to the CC lines.)

Although the genome of each CC line receives a balanced contribution from each founder strain, the founder contribution is not uniformly distributed along the genome because of the small number of recombination events undergone by each CC line. The genome of a CC line is essentially a mosaic of a small number of founder haplotype segments. On average, Pre-CC autosomal genomes had 142.3 segments on average (SD = 21.8) with a median segment length of 10.46 Mb (Aylor *et al.* 2011). As a result, some local subpopulation structure may be observed because the eight founder strains are not equally distant from each other (*i.e.,* three of founders are wild strains). The subpopulation structure is visible at the chromosome level. For example, there are several deep branches in the phylogeny tree of the selected PreCC mice built on Chromosome 10 (Figure 3B). The corresponding kinship matrix in Figure 4B shows that there are at least three subpopulations. The subpopulation structure is more evident if we narrow down to a 20 Mbps interval from 85 Mbps to 105 Mbps on Chromosome 10. The phylogeny tree in Figure 3C becomes more skewed, and the corresponding kinship matrix in Figure 4C also exhibits more pronounced structural patterns.

### Selected methods for comparison

We compare our algorithm HTreeQA with existing methods: TreeQA (Pan *et al.* 2008, 2009), BLOSSOC (Mailund *et al.* 2006; Besenbacher *et al.* 2009), EMMA (Kang *et al.* 2008), and HAM (Mcclurg *et al.* 2006) using both real and simulated data sets. Some other methods, such as HapMiner (Li and Jiang 2005) and TreeLD (Zöllner and Pritchard 2005), are too slow to process large data sets. For comparison purposes, we also implemented two other methods: SMA (single marker association mapping) and HAM (haplotype association mapping). In SMA, each SNP marker partitions samples into groups on the basis of the alleles. Analysis of variance is used to evaluate the significance of the partition. In HAM, a sliding window of three consecutive SNP is used to group samples on the basis of their sequences, and an analysis of variance is conducted to test the association between the phenotypes and the grouping. FastPhase (Scheet and Stephens 2006) is used to reconstruct haplotypes from the genotypes for the methods that require haplotype data (TreeQA and BLOSSOC).

Note that BLOSSOC, TreeQA, and HTreeQA are phylogeny-based methods. SMA, HAM, and EMMA are nonphylogeny-based methods. Although EMMA offers an option to use global phylogeny to estimate the kinship matrix, it does not test the associations between the phenotype and the phylogenetic trees. Table 2 shows the selected methods for comparison.

**Table 2:** Selected methods for comparison

	Methods
Nonphylogeny-based methods	SMA, HAM, EMMA
Phylogeny-based methods	BLOSSOC, TreeQA, HTreeQA

### Performance comparison on the white head spot phenotype

**Figure 5:**
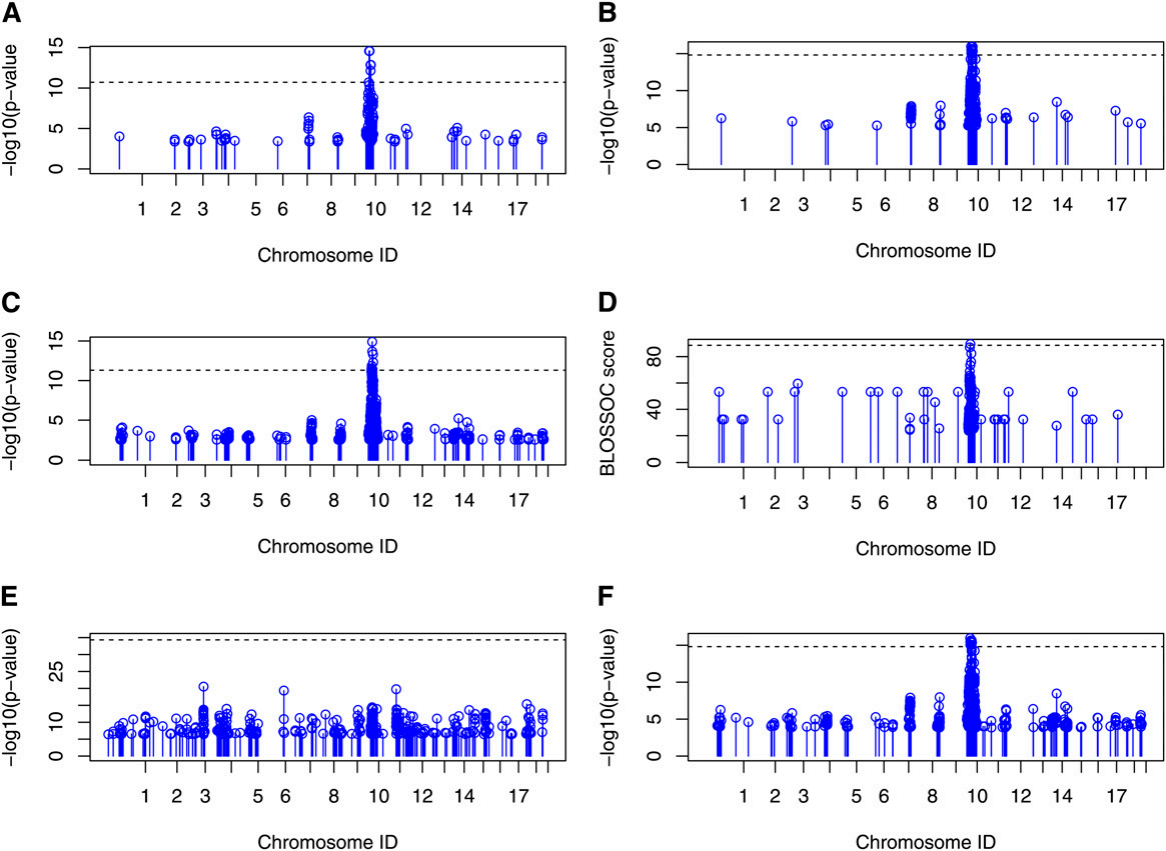
QTL mapping of the white head spot phenotype. Only the SNPs that have top 0.5% -log(p-value) or BLOSSOC score are plotted. One QTL is detected by HTreeQA, which is near the location of gene *kit ligand*. The remaining methods except HAM have similar results to that of HTreeQA. The dashed line is the significance level with FWER = 0.05. (A) Result from HTreeQA. (B) Result from TreeQA. (C) Result from EMMA. (D) Result from BLOSSOC. (E) Result from HAM. (F) Result from SMA.

The white head spot is known as a recessive trait carried by WSB/EiJ (Aylor *et al.* 2011). We apply the selected methods to the white head spot phenotype. A permutation test is applied to control the FWER (Westfall and Young 1993, Churchill and Doerge 1994). With FWER = 0.05, all the selected methods except HAM identify a QTL, which is approximately 100M bps in Chromosome 10 (Figure 5). This QTL is close to a gene named *kit ligand* known to be controlling white spotting (Aylor *et al.* 2011). HAM fails to detect the QTL because it does not consider the compatibility between consecutive SNPs. The incompatibility between two consecutive SNPs suggests a high possibility of having a historical recombination event between them. Treating an interval containing incompatible SNPs as a single locus may lead to spurious results. The phylogeny-based methods, including HTreeQA, can avoid this problem by only examining phylogeny trees constructed from compatible intervals.

In each panel of Figure 3, A−D, the nearest common ancestor of the four white head spot mice (highlighted in red) is marked by a circled “A.” We observe from Figure 3, A−C that the distance between the common ancestor and the four mice becomes smaller when the interval on which the tree is built becomes shorter. It is evident that the four white spot mice are clustered in the phylogeny tree built over the 20 Mb region in Figure 3C, despite the local population structure. This becomes clearer in Figure 3D, where the four white head spot mice having white head spot located on the same branch of the tristate semi-perfect phylogeny tree built on the compatible interval at the QTL. This demonstrates the effectiveness of the proposed model.

### Performance comparison on the mouse running distance phenotype

**Figure 6:**
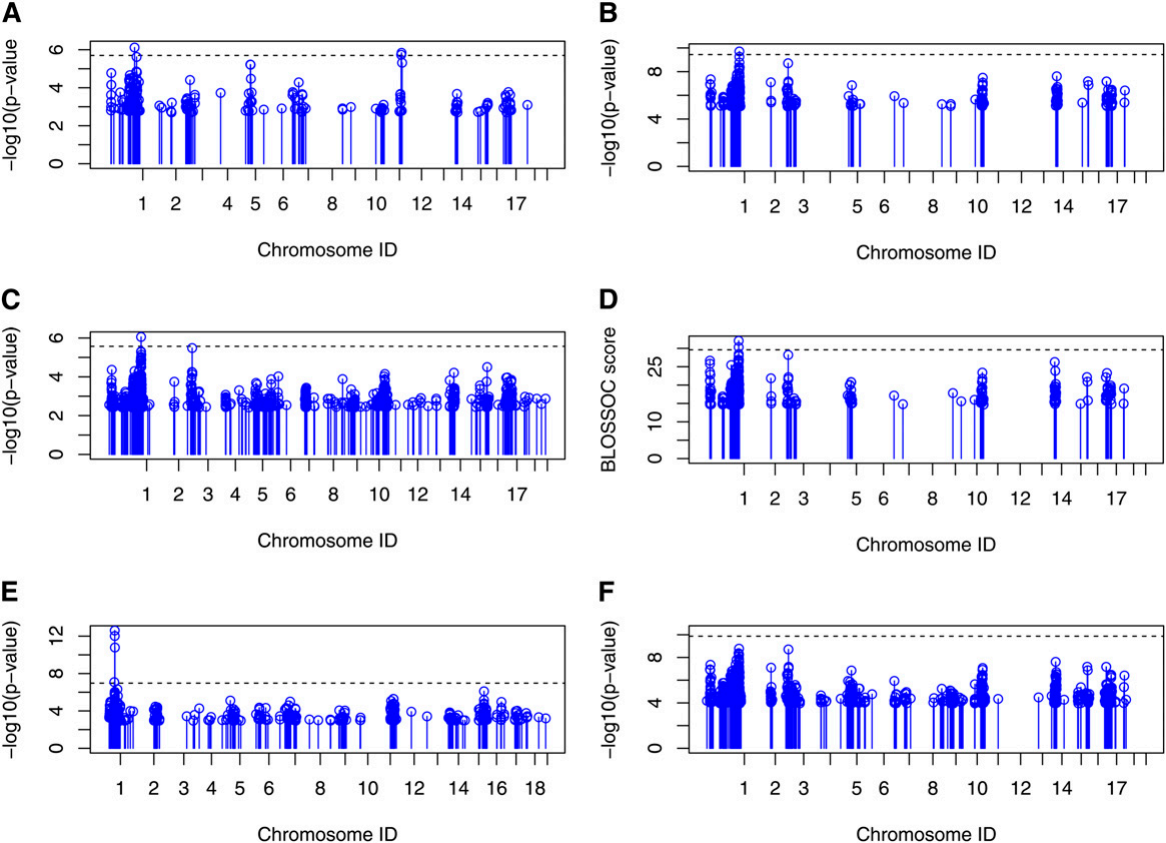
QTL for mice daily average running distance. Only the SNPs that have top 0.5% -log(p-value) or BLOSSOC score are plotted in the figure. The dashed line is the significance level with FWER = 0.05. (A) Result from HTreeQA. (B) Result from TreeQA. (C) Result from EMMA. (D) Result from BLOSSOC. (E) Result from HAM. (F) Result from SMA.

We apply the selected methods on the phenotype “Mouse Running Distance at day 5/6.” With FWER = 0.05, all the methods except SMA identified a QTL at 169 to 169.2 Mbp (89 cM) on Chromosome 1 as shown in Figure 6. The QTL falls into the previously reported *cplaq3* region (Mayeda and Hofstetter 1999). A later study also confirmed this QTL (Hofstetter *et al.* 2003).

Among the selected methods, only HTreeQA identified another QTL with FWER = 0.05, in the region of 16 M to 25 Mbps (8-12.5 cM) on Chromosome 12. The QTL falls into an unnamed QTL region at 11 cM on Chromosome 12 reported in (Hofstetter *et al.* 2003). The reason that many methods fail to report this QTL is that these methods have limited power in detecting non-additive effects. This result demonstrates that HTreeQA can detect more types of effects than the other methods.

### Simulation study

To examine the performance of HTreeQA in a controlled environment, we simulated three different types of effects: additive, recessive, and overdominant. For each selected method, only the SNPs with significance level FWER = 0.05 are reported as QTL. Because we remove the causative SNPs in the simulated data before we run QTL analysis, to measure the accuracy of the result, we considered a reported QTL a true positive when it was located within 50 SNPs from the causative SNP. We used three measurements to estimate the performance of each method: *precision*, *recall*, and *F1 score*. Precision is defined as the ratio between the number of true QTL that are detected and the total number of detected QTL. Recall is defined as the ratio between the number of true QTL that are detected and the total number of true QTL that are simulated. The F1 score is the harmonic mean of precision rate and recall rate, and is defined as follows:

$$F1=\frac{2\text{×}Precision\text{×}Recall}{Precision+Recall}.$$

**Figure 7:**
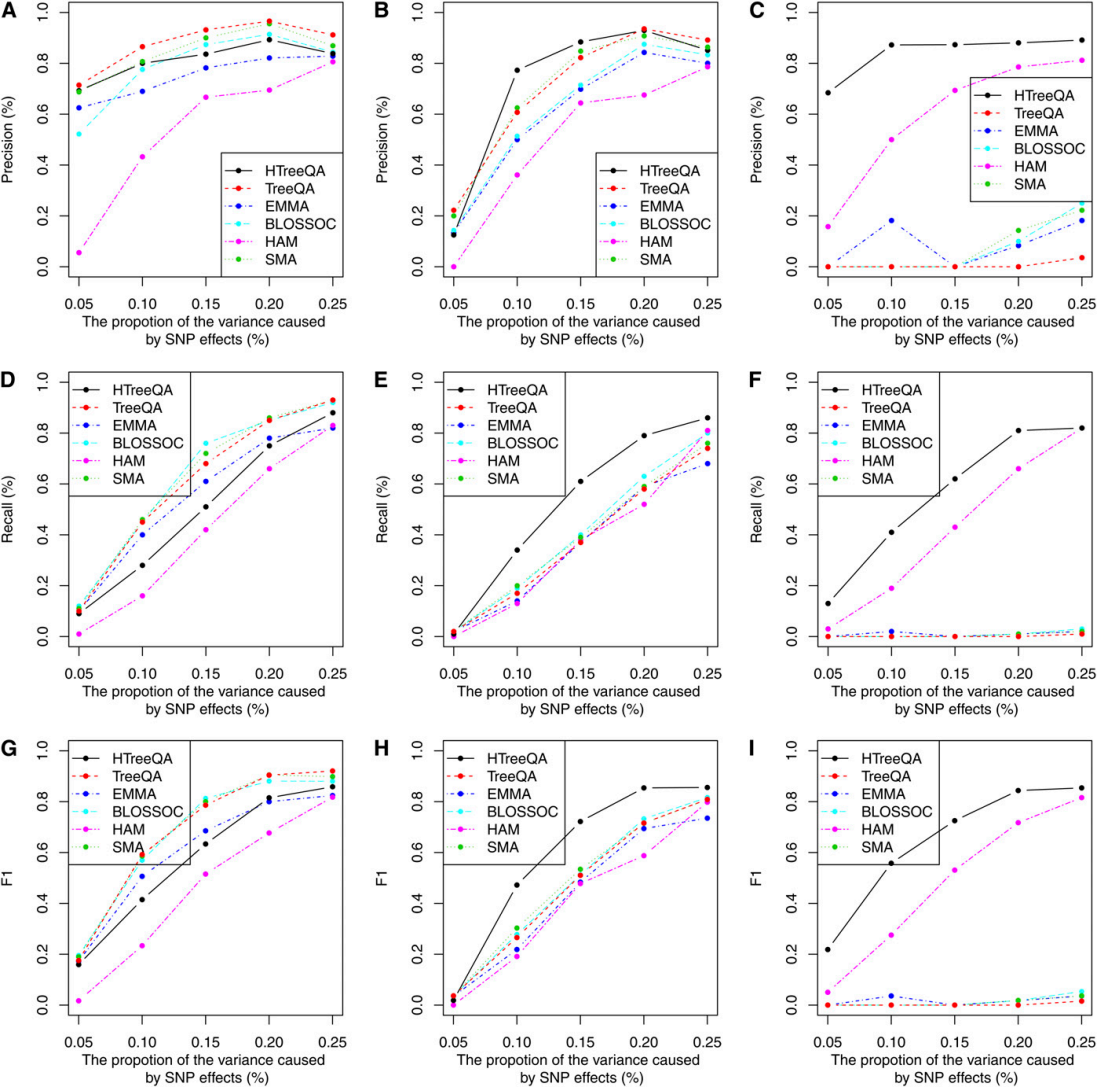
Comparison of HTreeQA, TreeQA, SSA, BLOSSOC, EMMA, and HAM under different genetic models. (A), (D), and (G) are under additive models; (B), (E), and (H) are under recessive models; (C), (F), and (I) are under overdominant models.

Figure 7 compares selected methods. HTreeQA shows comparable performance to that of other methods in the additive model. In the recessive model and the overdominant model, HTreeQA demonstrates significant advantage over other methods. Because HTreeQA does not have any assumption of the type of genetic effect, it offers consistent power for detecting any effect. Other methods except HAM implicitly assume the additive model.

The phasing step required by the phylogeny-based methods BLOSSOC and TreeQA (for handling heterozygosity) will impair their ability in detecting associations between the phylogeny and the phenotype. The extent of its effect varies for different genetic models, especially with regard to heterozygous samples. It affects the additive model the least and overdominant model the most. For a homozygous sample, the nodes corresponding to the two haplotypes carry the same allele, and thus their phenotypes always belong to the same allele group. This may cause minor inflation of the QTL signals because the two haplotypes are treated as independent samples by these methods. For a heterozygous sample the two haplotypes carry different alleles and therefore their corresponding nodes and phenotype are in two allele groups. Under the additive model assumption, one allele group contains all homozygous samples with high phenotype values, and the other contains all homozygous samples with low phenotype values. The heterozygous samples have medium phenotype values, which are added to both allele groups. This may cause minor deflation of the QTL signals. This is why all selected methods have comparable performance. TreeQA slightly outperforms others because its local phylogeny trees can well model the local population structure and separate QTL signals from genetic background.

However, under the assumption of overdominant model, heterozygous samples may have extreme phenotype values (beyond the range of phenotype values of the homozygous samples). These extreme phenotype values will always be in both allele groups; therefore, the phylogeny representation for phased data cannot explain the overdominant effects at all. This is why the traditional phylogeny-based methods like BLOSSOC and TreeQA fail under such a model. Note that HTreeQA does not require phasing. The tristate semi-perfect phylogeny tree has a partition that separates the heterozygous samples from the homozygous samples and thus it is able to detect an overdominant effect. Under the recessive model assumption, the heterozygous allele carries the same effect as one of the two homozygous alleles. Thus, the impact of assigning haplotypes of the heterozygous samples to the two allele groups is greater than that under the additive model and is not as great as that under the overdominant model. Again, this does not affect HTreeQA. Overall, HTreeQA has the best performance in recessive models and overdominant models.

### Running time comparison

We present the running time for each selected method on a machine with Intel i7 2.67-GHz CPU and 8-G memory. We tested all methods using a dataset containing 180K SNPs and 184 individuals. Table 3 shows the running time of these methods. If phasing is required, this step usually takes more than 40 hr and dominates the running time. HTreeQA demonstrates a great advantage by completely avoiding haplotype reconstruction. It is more than 600 times faster than the other methods that require haplotype data. HTreeQA is 15 times faster than EMMA because it does not need to explicitly incorporate the effect of global population structure as EMMA does. The running time of HTreeQA is comparable with that of SMA and HAM, the simplest models for QTL studies. They are not as effective as HTreeQA, as demonstrated in the real phenotype and simulation studies.

**Table 3:** Running time comparison of the selected methods

Methods	Running Time	Require Haplotype Reconstruction?
SMA	10 min	No
BLOSSOC	40 hr	Yes
HAM	20 min	No
TreeQA	40 hr	Yes
EMMA	3 hr 20 min	No
HTreeQA	12 min	No

The running time is measured on a machine with Intel i7 2.67-GHz CPU and 8-G memory.

### The choice between HTreeQA, TreeQA, and EMMA

HTreeQA is proven to have an overall lower error rate than TreeQA and other similar approaches (in Appendix 1). It can handle heterozygous genotype properly. It is suitable for genome-wide association studies on any populations, including the incipient CC lines, Heterogeneous Stock, Diversity Outbred, and Recombinant Inbred Crosses of CC lines. TreeQA is the best choice if one focuses on the additive effects. EMMA can correct for global population structure but is not able to address any local population structure. It degenerates to a simple linear model when applied to CC population with an evenly distributed global population structure as shown in Appendix 2. This represents a limitation of EMMA because local population structures exist in every mammalian resource, even though we only show the results on the CC population in this article.

## Conclusions

We propose a novel approach for local phylogeny-based QTL mapping on genotype data without haplotype reconstruction. We analyze the incipient CC and show that there is no significant global population structure but visible local population structure. Such local population structure may bias the QTL mapping if it is not addressed properly. The notion of a tristate semi-perfect phylogeny tree is introduced to represent accurate genetic relationships between samples in short genomic regions. As a generalization of the perfect phylogeny tree (defined on haplotypes), a tristate semi-perfect phylogeny tree treats the heterozygous allele as the third state. It provides the power of modeling a wide range of genetic effects and delivers unbiased and consistent performance. It also guarantees a lower theoretical error rate of statistical tests than the perfect phylogeny based approach. This is a significant advantage over any previous methods that have strong bias toward an additive model. It is also worth noting that HTreeQA is much more computationally efficient than any alternative approach.

## Supplementary Material

Supporting Information

## Appendix 1

### Theoretical Analysis on HTreeQA and TreeQA

In this section, we present the theoretical analysis of HTreeQA and TreeQA under different genetic models. It can be shown that HTreeQA has a theoretical advantage over the general phylogeny-based approach using phased haplotypes. We first prove that testing single SNPs on genotypes data has lower error rate than on phased haplotype data. We then analyze its potential effect on these two different phylogeny approaches.

We assume that the causative SNP contains *n*_1_ homozygous subjects, *n_h_* heterozygous subjects, and *n*_0_ homozygous wild subjects. We also assume that the phenotypes can be approximated by a normal distribution, which is a reasonable assumption in most cases. We use *X_i_*_1_, *X_ih_*, and *X_i_*_0_ to model each subject in these three groups:

$$X_{i1}∼N\left(1,\varphi_{1}\right)$$

$$X_{i0}∼N\left(0,\varphi_{0}\right)$$

$$X_{ih}∼N\left(m_{h},\varphi_{h}\right)$$

Without loss of generality, we assume the samples are independent and follow three normal distributions with different means and variances for each group. If *m_h_* equals 0 or 1, it is a recessive model. If *m_h_* is between 0 and 1, it is an additive model. Otherwise it is an overdominance model. If we use a phylogeny-based approach on phased haplotypes, each homozygous subject has a duplicate homozygous subject, and each heterozygous subject is treated as two different homozygous subjects. Thus we could use two groups to represent the partition of this SNP, {*X*_11_, …, $X_{n_{1}1}$, *X*_11_, …, $X_{n_{1}1}$, *X*_1*h*,_ …, $X_{n_{h}h}$} and {*X*_10_, …, $X_{n_{h}h}$, *X*_10_, …, $X_{n_{0}0}$, *X*_1*h*,_ …, $X_{n_{h}h}$}. If we use HTreeQA, which is directly applied on genotype data, there are three groups based on the allele of each subject, {*X*_11_, …, $X_{n_{1}1}$}, {*X*_1*h*,_ …, $X_{n_{h}h}$}, and {*X*_10_, …, $X_{n_{0}0}$}.

(A1) $${\bar{X}}_{1}=\frac{\sum_{i=1}^{n_{1}}X_{i1}}{n_{1}}$$

(A2) $${\overset{-}{X}}_{0}=\frac{\sum_{i=1}^{n_{0}}X_{i0}}{n_{0}}$$

(A3) $${\overset{-}{X}}_{h}=\frac{\sum_{i=1}^{n_{h}}X_{ih}}{n_{h}}$$

(A4) $${\overset{-}{X^{\prime }}}_{1}\frac{2\sum_{i=1}^{n_{1}}X_{i1}+\sum_{i=1}^{n_{h}}X_{ih}}{2n_{1}+n_{h}}$$

(A5) $${\overset{-}{X^{\prime }}}_{0}\frac{2\sum_{i=1}^{n_{0}}X_{i0}+\sum_{i=1}^{n_{h}}X_{ih}}{2n_{0}+n_{h}}$$

(A6) $$\overset{-}{X}=\frac{\sum_{i=1}^{n_{1}}X_{i1}+\sum_{i=1}^{n_{0}}X_{i0}+\sum_{i=1}^{n_{h}}X_{ih}}{n_{1}+n_{0}+n_{h}}$$

(A7) $$\begin{array}{l} S_{Haplotype}=2n_{1}{\left({\overset{-}{X^{\prime }}}_{1}-\overset{-}{X}\right)}^{2}+n_{h}{\left({\overset{-}{X^{\prime }}}_{1}-\overset{-}{X}\right)}^{2}\\ \;+n_{h}{\left({\overset{-}{X^{\prime }}}_{0}-\overset{-}{X}\right)}^{2}+2n_{0}{\left({\overset{-}{X^{\prime }}}_{0}-\overset{-}{X}\right)}^{2} \end{array}$$

(A8) $$\begin{array}{l} T_{Haplotype}=2\overset{n_{1}}{\underset{i=1}{\sum }}{\left(X_{i1}-{\overset{-}{X^{\prime }}}_{1}\right)}^{2}+\overset{n_{h}}{\underset{i=1}{\sum }}{\left(X_{ih}-{\overset{-}{X^{\prime }}}_{1}\right)}^{2}\\ +\overset{n_{h}}{\underset{i=1}{\sum }}{\left(X_{ih}-{\overset{-}{X^{\prime }}}_{0}\right)}^{2}+2\overset{n_{0}}{\underset{i=1}{\sum }}{\left(X_{i0}-{\overset{-}{X^{\prime }}}_{0}\right)}^{2} \end{array}$$

(A9) $$\begin{array}{l} S_{Genotype}=n_{1}{\left({\overset{-}{X}}_{1}-\overset{-}{X}\right)}^{2}+n_{h}{\left({\overset{-}{X}}_{h}-\overset{-}{X}\right)}^{2}\\ \;+n_{0}{\left({\overset{-}{X}}_{0}-\overset{-}{X}\right)}^{2} \end{array}$$

(A10) $$\begin{array}{l} T_{Genotype}\overset{n_{1}}{\underset{i=1}{\sum }}{\left(X_{i1}-{\overset{¯}{X}}_{1}\right)}^{2}+\overset{n_{h}}{\underset{i=1}{\sum }}{\left(X_{i1}-\overset{¯}{X}\right)}^{2}\\ +\overset{n_{0}}{\underset{i=1}{\sum }}{\left(X_{i1}-{\overset{¯}{X}}_{0}\right)}^{2} \end{array}$$

Following Equation 1 in the *Methods* and A1 to A10, we define *F_Haplotype_* and *F_Genotype_* to represent the *F*-statistics of these two different groupings respectively.

$$F_{Haplotype}=\frac{S_{Haplotype}}{T_{Haplotype}}$$

$$F_{Genotype}=\frac{S_{Genotype}}{T_{Genotype}}$$

For the following analysis we assume that *n*_1_, *n_h_*, and *n*_0_ are large numbers, and we use ‘*a* ∼ *b*’ to denote *a* and *b* are asymptotically equal when the sample size approaches infinity. Here *b* is a number instead of a distribution. Similarly, we use ‘≲’ and ‘≳’ to represent asymptotically less than and greater than relationship respectively. Next, we prove that directly testing associations between a phenotype and the genotypes has a lower error rate than testing the association between the phenotypes and phased haplotypes when the sample size is large.

First, for large sample sizes, we have the following lemmas as an immediate consequence of the Weak Law of Large Number Theorem,

### Lemma 1

$$\begin{array}{l} {\overset{-}{X}}_{1}∼1{\overset{-}{X}}_{0}∼0\\ {\overset{-}{X}}_{h}∼m_{h}{\overset{-}{X^{\prime }}}_{1}∼\frac{2n_{1}+m_{h}}{2n_{1}+n_{h}}\\ {\overset{-}{X^{\prime }}}_{0}∼\frac{m_{h}}{2n_{0}+n_{h}}\;\;\;\overset{-}{X}∼\frac{n_{1}+m_{h}n_{h}}{n_{1}+n_{0}+n_{h}} \end{array}$$

### Lemma 2 S_*Haplotype*_ ≲ 2S_*Genotype*_

#### Proof

Sketch: The asymptotic values for variables in Equations A7 and A9 are determined by Lemma 1. And the expanded form of *S_Haplotype_* − *2S_Genotype_* is a quadratic function of *m_h_*_,_ and its discriminant is smaller than 0.

### Lemma 3

*N random variables Y_i_* *are independent and identically distributed, with mean value μ and finite variance ϕ. For any real number γ ≠ μ, when N → ∞, we have*

(A11) $$P\left(\overset{N}{\underset{i=1}{\sum }}{\left(Y_{i}-\gamma \right)}^{2}>\overset{N}{\underset{i=1}{\sum }}{\left(Y_{i}-\mu \right)}^{2}\right)\to 1.$$

#### Proof

Without loss of generality, we assume μ − γ > 0,

$$\begin{array}{l} P\left(\overset{N}{\underset{i=1}{\sum }}{\left(Y_{i}-\gamma \right)}^{2}>\overset{N}{\underset{i=1}{\sum }}{\left(Y_{i}-\mu \right)}^{2}\right)\\ =P(\overset{N}{\underset{i=1}{\sum }}{\left(Y_{i}-\mu \right)}^{2}+2\overset{N}{\underset{i=1}{\sum }}\left(Y_{i}-\mu \right)\left(\mu -\gamma \right)+\overset{N}{\underset{i=1}{\sum }}{\left(\mu -\gamma \right)}^{2}\\ >\overset{N}{\underset{i=1}{\sum }}{\left(Y_{i}-\mu \right)}^{2})\\ =P\left(\overset{N}{\underset{i=1}{\sum }}Y_{i}-n\mu >-N\left(\mu -\gamma \right)/2\right)\\ t=P\left(\frac{\sum_{i=1}^{N}Y_{i}-n\mu }{\sqrt{N}\varphi }>-\frac{\sqrt{N}\left(\mu -\gamma \right)}{2\varphi }\right)\\ =1-\text{Φ}\left(-\frac{\sqrt{N}\left(\mu -\gamma \right)}{2\varphi }\right)\left(Central\;Limit\;Theorem\right)\\ \to 1\;\left(n\to \infty \right) \end{array}$$

### Lemma 4 T_*Haplotype*_ ≲ 2T_*Genotype*_

#### Proof

${\overset{-}{X}}_{1}$, ${\overset{-}{X}}_{0}$ and ${\overset{-}{X}}_{h}$ converge to the mean of *X_i_*_1_, *X_i_*_0_ and *X_ih_* by Lemma 1, but ${\overset{-}{X^{\prime }}}_{1}$ and ${\overset{-}{X^{\prime }}}_{0}$ converge to two different values as shown in Lemma 1. Lemma 4 follows directly from Lemma 3.

### Theorem 2 *F_Haplotype_* ≲* F_Genotype_*

#### Proof

This can be directly proved from Lemmas 2 and 4.

We use *F_Null_* to represent the statistics of testing non-causative partitions from either a semi-perfect phylogeny tree or a perfect phylogeny tree. Because phenotype values can be approximated by a normal distribution, the distributions of *F_Null_* using these two approaches converge to the same distribution. Although it is unlike that the causative SNP is genotyped in real situation, by linkage disequilibrium, there exists a partition in the semi-perfect phylogeny tree or the perfect phylogeny tree based on neighboring SNPs that is very similar to the partition of the causative SNP. Therefore, we have the following theorem.

### Theorem 3 P(F_*Null*_ > F_*Haplotype*_) ≳ P(F_*Null*_ > F_*Genotype*_)

The probabilities in the Theorem 3 are the error rates of TreeQA on phased haplotypes and HTreeQA on genotypes.

## Appendix 2

### EMMA will degenerate to standard linear model in Collaborative Cross

First, we define a new class of matrix named *K_uniform_*(*D*, *S*),

(A12) $$K_{\mathrm{uniform}}\left(D,S\right)=\left(\begin{array}{cccc} D & S & \cdots & S\\ S & D & \cdots & S\\ \vdots & \vdots & \ddots & \vdots \\ S & S & \ldots & D \end{array}\right)$$

where D represents the diagonal entries and S represents the off-diagonal entries in the matrix.

Assume that **y** is a vector of phenotypes, **X** is a vector of fixed effects from a SNP, and **e** is a vector of residual effects for each individual. We omit the indicator matrix *Z* used in original EMMA model, because in the CC data, *Z* is an identity matrix. The EMMA model is presented in the following form:

(A13) y=μ1+Xβ+u+e

(A14) $$u∼MVN\left(0,\sigma_{K}^{2}K_{emma}\right)$$

(A15) $$\mu ∼Norm\left(0,\sigma_{\mu }^{2}\right)$$

(A16) $$e∼MVN\left(0,\sigma_{e}^{2}K_{uniform}\left(1,0\right)\right)$$

where *MVN* represents a multivariate normal distribution. *K_emma_* is the kinship matrix inferred by the EMMA package.

Similarly, a standard linear model is in the following form:

(A17) y=μ1+Xβ+e

(A18) $$\mu ∼Norm\left(0,\sigma_{\mu }^{2}\right)$$

(A19) $$e∼MVN\left(0,\sigma_{e}^{2}K_{uniform}\left(1,0\right)\right)$$

Assuming the samples of a population have exactly the same relatedness *S*:

(A20) $$K_{uniform}\left(1,S\right)=K_{uniform}\left(S,S\right)+K_{uniform}\left(1-S,0\right)$$

(A21) $$\mu 1∼MVN\left(0,\sigma_{\mu }K_{uniform}\left(1,1\right)\right)$$

(A22) $$e∼MVN\left(0,\sigma_{\mu }K_{uniform}\left(1,0\right)\right)$$

Thus, if *K_emma_* = *K_uniform_*(1, *S*), by re-factorization of the random effects in the EMMA model, we have

(A23) y=μ1+Xβ+e

(A24) $$\mu 1∼MVN\left(0,K_{uniform}\left(\sigma_{\mu }^{2}+\sigma_{K}^{2}S,\sigma_{\mu }^{2}+\sigma_{K}^{2}S\right)\right)$$

(A25) $$e∼MVN\left(0,\sigma_{e}^{2}K_{uniform}\left(\left(1-\sigma_{K}^{2}\right)S+1,0\right)\right)$$

This has the same form of a standard linear regression model. In CC, the kinship matrix can be represented by a *K_uniform_* matrix with tolerable numerical error. This suggests that there is no significant difference between EMMA and the standard linear regression model when these two methods are applied to Collaborative Cross data.
