# Supplementary material for: HTreeQA: Using Semi-Perfect Phylogeny Trees in Quantitative Trait Loci Study on Genotype Data
Source: G3 (Bethesda). 2012 Feb 1;2(2):175–89. doi: 10.1534/g3.111.001768 (PMC3284325; doi:10.1534/g3.111.001768)
Supplement: Supporting Information [file supp_2_2_175__index.html]

Supporting Information 

# HTreeQA: Using Semi-Perfect Phylogeny Trees in Quantitative Trait Loci Study on Genotype Data

## Supporting Information for Z. Zhang, X. Zhang, and W. Wang, 2012

**Files in this Data Supplement:**

- File S1 - Supporting data (.zip, 2 KB)
